# Gender and water, sanitation, and hygiene: Three opportunities to build from recent reporting on global progress, 2000–2022

**DOI:** 10.1371/journal.pmed.1004297

**Published:** 2023-10-18

**Authors:** Juliet Willetts, Jess MacArthur, Naomi Carrard

**Affiliations:** University of Technology Sydney, Institute for Sustainable Futures, Sydney, Australia

## Abstract

In this Perspective, Juliet Willetts and colleagues discuss opportunities to stimulate progress in gender-related aspects of water, sanitation and hygiene.

In July 2023, the Joint Monitoring Programme (JMP) released a progress report on household drinking water, sanitation, and hygiene (WASH) (2000–2022) with a special focus on gender [1]. The report presents international comparable estimates of WASH access in households at national, regional, and global levels and extends this analysis to address gendered aspects where data allow. In this Perspective, we commend this effort and highlight 3 opportunities we believe could stimulate further progress.

The focus on gender is welcome. As stated in the JMP report, progress on WASH is widely recognised to influence achievement of Sustainable Development Goal (SDG) 5 to “realize gender equality and empower all women and girls” [1]. Equally, there is broad agreement that gender inequalities pose challenges for realisation of the SDG 6 targets on WASH and that poor progress places different and unequal burdens on women, men, girls, and boys. Fortunately, there has been much progress in the last decade since we first published on the need to promote synergies across the gender and WASH Millennium Development Goals [2] building on the work of others advocating about these issues since the 1980s [3].

The JMP report highlights a growing emphasis on gender–WASH connections, providing valuable insights into the interlinkages between gender and WASH through the analysis of available national statistics. The report includes new data on menstrual health, shedding light on how 2 billion women of reproductive age meet their needs, while also considering the impact of wealth, gender, age, geography, and disability. The report also addresses long-standing debates on aspects of gender and WASH [4], for instance, making clear that women, men, boys, and girls—depending on the context—may all suffer from the burden of water carriage, albeit with women and girls suffering more overall. For sanitation, although national sex-disaggregated data are not available, the report brings together data on shared sanitation and on women’s feelings of safety walking alone at night, providing insight into specific gendered impacts of sanitation access.

The first opportunity to build on global progress on gender and WASH relates to framing—moving away from an instrumental orientation towards transformative change. An instrumental orientation is defined as one which accepts and leverages women’s traditional gender roles and norms towards improved WASH outcomes, whereas transformative change involves challenging such roles and norms. While there is recognition of a two-way interrelationship between gender equality and WASH, the JMP report tends to be framed around “how addressing gender inequalities can accelerate progress on WASH” [1]. At times, the report falls into the trap of an instrumental focus on gender, which could serve to reinforce rather than challenge existing gender norms. For instance, the report points out: “Lack of handwashing facilities disproportionately impacts adolescent girls and women who are primarily responsible for child care and domestic chores in many countries around the world” [1]. This latter dynamic is a core gender norm that ideally WASH behaviour change interventions should challenge rather than accept, as noted by authors examining targeting of mothers in hygiene campaigns [5]. While the report clearly asserts the value of WASH for driving gender equality, for example, noting the well-established connections between improving the accessibility of drinking water and empowering women and girls, these links tend to focus on women’s practical needs rather than their strategic gender interests [6]. Strategic gender interests concern longer-term structural changes in society regarding social norms that govern the status and roles of women and other gender minorities, such as legislation for equal rights and increased participation in decision-making. As such, there is a missed opportunity to consider how approaches to strengthen WASH services might also challenge social norms that drive gender inequality.

A more radical agenda is needed if genuine contributions to SDG5 are to be achieved through WASH progress and to realise the potential for transformed gender roles, norms, and dynamics, known as gender transformation [7]. This would require the use of more recent frameworks on gender and WASH [8] that emphasise not only the categories of gender integration, but also their differing motivations such as welfare, equity, efficiency, empowerment, and equality. This is a much-needed approach, as evidenced by a recent systematic review that found that only 7% of studies on gender and WASH had a clear definition or conceptualisation of empowerment [9]. What is positive, however, is the language of “gender equality” used throughout the JMP report and the reference to both women and men. Too often, a focus on gender can inadvertently be reduced back to a singular focus on women, which can place the burden to drive changes towards greater equality on women (who must be “empowered”), rather than being the responsibility of both men and women to remove societal barriers to women.

A second opportunity is finding solutions to gain insight beyond the household level—a challenge that is explicitly recognised in the report. As stated above, JMP relies on nationally available statistics and, for WASH, these are predominantly in the form of 5-yearly household surveys (UNICEF’s Multiple Indicator Cluster Survey (MICS)). Household surveys tend to render intrahousehold dynamics invisible. However, JMP’s consultation process to expand gender-related indicators has been an important advance. Upcoming MICS surveys will include additional standardised indicators related to menstruation and attitudes to gender-based violence, which will add important new insights. Taking this further, there could be opportunity in future to include questions on decision-making, similar to questions covered in the Demographic Health Survey (DHS), or to integrate these datasets. Given that the survey protocol for MICS already involves speaking separately to women, men, and adolescents in household-focused interviews, there is the potential in future to also integrate questions about intrahousehold access or use of WASH services.

The third opportunity concerns the users of JMP data, with a call to interpret and use the data carefully and employ complementary methods to understand gender dynamics. The large-scale, nationally representative data presented by JMP provide an important source that is powerful for making arguments for resource allocation and for tracking global progress. However, large-scale, quantitative datasets are necessarily reductive, and the use of complementary sources and methods is critical for understanding and progressing gender equality and WASH within different country contexts. Other quantitative and qualitative tools are increasingly available [10–12]. Use of these and other methods can provide critical complementary information about the complex social change processes required to change gender dynamics, can track unintended outcomes, and thereby mitigate potential harm from well-intentioned efforts that inadvertently cause backlash and resistance.

In conclusion, we appreciate and admire the advances that the JMP report displays in addressing and illuminating aspects of gender and WASH. The scale of the WASH challenge remains substantial, with 2.2 billion people still lacking safe drinking water, 3.5 billion lacking safely managed sanitation, and the 2 billion lacking basic hygiene services [1]. The gender equality challenge is no smaller. Global reporting in 2022 notes that, at current rates, it may take another 286 years to remove discriminatory laws and close prevailing gaps in legal protections for women and girls [13]. As efforts are bolstered to progress safe WASH services and gender equality, we challenge readers to go further. To build on global progress on WASH, it is important to frame gender-related issues carefully, to move beyond instrumental intentions that prioritises WASH-related outcomes towards genuine gender transformation, and to hold on to the need to pursue gender and WASH in mutually synergistic ways. Equally, advocacy is needed for nationally representative surveys that incorporate intrahousehold data. Lastly, users of global data are urged to complement these data with other quantitative and qualitative monitoring and research tools to navigate pathways to improved WASH and gender equality. Together, it is possible to accelerate progress towards universal access to water and sanitation and at the same time proactively contribute to much-needed efforts to advance gender equality across the globe.

## Abbreviations

DHS: Demographic Health Survey
JMP: Joint Monitoring Programme
MICS: Multiple Indicator Cluster Survey
SDG: Sustainable Development Goal
WASH: water, sanitation, and hygiene
